# Antidepressant drugs modulate growth factors in cultured cells

**DOI:** 10.1186/1471-2210-8-6

**Published:** 2008-03-04

**Authors:** Andreas W Henkel, Wolfgang Sperling, Andrea Rotter, Udo Reulbach, Cornelia Reichardt, Dominikus Bönsch, Juan M Maler, Johannes Kornhuber, Jens Wiltfang

**Affiliations:** 1 Department of Psychiatry and Psychotherapy, University of Erlangen-Nuremberg, Schwabachanlage 6, 91054 Erlangen, Germany

## Abstract

**Background:**

Different classes of antidepressant drugs are used as a treatment for depression by activating the catecholinergic system. In addition, depression has been associated with decrease of growth factors, which causes insufficient axonal sprouting and reduced neuronal damage repair. In this study, antidepressant treatments are analyzed in a cell culture system, to study the modulation of growth factors.

**Results:**

We quantified the transcription of several growth factors in three cell lines after application of antidepressant drugs by real time polymerase chain reaction. Antidepressant drugs counteracted against phorbolester-induced deregulation of growth factors in PMA-differentiated neuronal SY5Y cells. We also found indications in a pilot experiment that magnetic stimulation could possibly modify BDNF in the cell culture system.

**Conclusion:**

The antidepressant effects antidepressant drugs might be explained by selective modulation of growth factors, which subsequently affects neuronal plasticity.

## Background

The mechanism of action of antidepressant drugs is far from well understood. The "catecholamine deficiency hypothesis" stated that affective disorders like depression originate in pathologic reduction of catecholamine levels in the brain [1].

The beneficial effects of antidepression treatment on serotonin and dopamine turnover and the associated receptors start a few weeks after onset of therapy, casting doubt on a simple mode of action, which should be effective much faster. Other classes of psychotrophic drugs, like benzodiazepine, which interact with neurotransmitter receptors too, become effective within minutes after drug administration. The mechanism of therapeutic latency of antidepressant and neuroleptic drugs is not clearly understood. Current hypotheses include slow adaptive processes after fast access to primary drug targets[2].

Alternatively, it was proposed that antidepressant drugs alter gene expression or induce nerve plasticity by rearranging neuronal connections [3]. Because the formation and modification of neuronal connections depend on nerve activity [4], it appears very likely that drugs trigger formation of new connections and strengthens already existing by enhancing exocytosis [5]. Recent work on exo- and endocytosis revealed profound effects of antidepressant drugs on neurotransmitter secretion and receptor cycling [6,7]. TGFβ2 occurs in astroglia and various neurons and is known to promote the effects of growth factors such as NT-3 and 4, GDNF, CNTF and bFGF [8]. Therefore up-regulation of TGFβ2 and it's secretion via regulated pathways by PC-12 cells and hippocampus neurons point towards indirect regulatory roles [9].

In addition to pharmacological treatments, rTMS was first introduced in 1985 [10] as a non-invasive tool of brain stimulation. It was proposed that rTMS might be a promising technique for the therapy of depression [11,12]. However, recent meta analyses and multi-center trials revealed that rTMS was not as effective as ECT, specifically for the short term treatment [13,14].

This study focuses mainly on the analysis of growth factor transcription by real time PCR analysis in a cell culture system, after administration of antidepressant drugs. Additionally, morphological effects of antidepressant treatment were analysed by microscopy and effects of magnetic stimulation on growth factors were also investigated in preliminary pilot experiments.

We hypothesize that antidepressant drugs rather modulate the expression of growth factors than just simply increase their expression, which in turn stabilize, enhance or even create neural connections between specific brain areas, involved in mood regulation.

## Results

### Antidepressant drugs change transcription of growth factors

**Figure 1:**
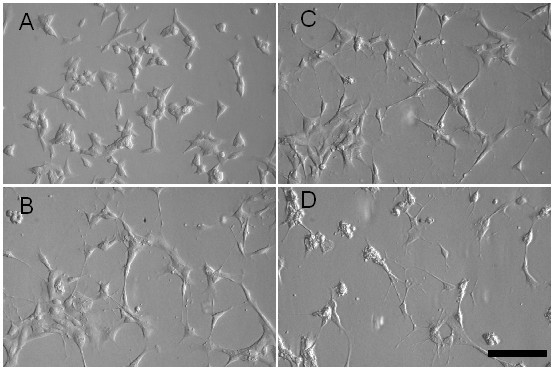
**Differentiation by PMA and treatment with anti-depressive drugs alter cell morphology**. **A. **Undifferentiated SY5Y cells; **B. **10 days treatment with PMA; **C. **14 days treatment with PMA and fluoxetine; **D. **14 days treatment with PMA and tranylcypromine. Scale bar = 20 μm.

**Figure 2:**
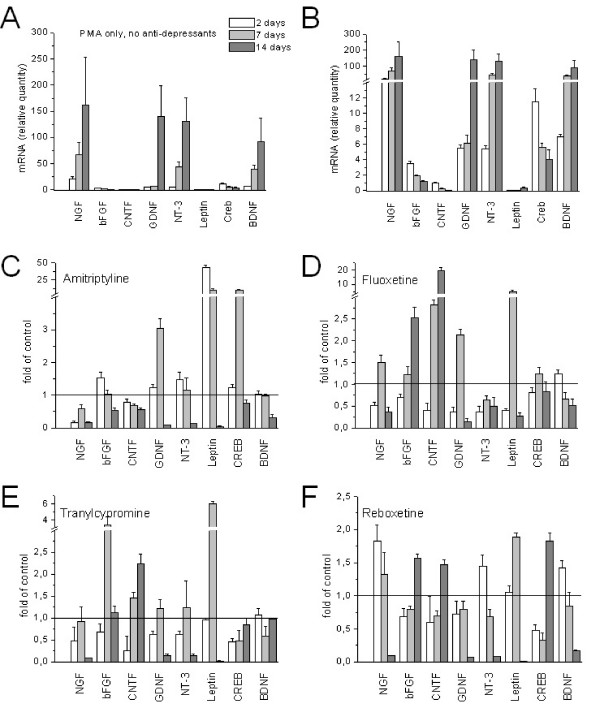
**Transcription of growth factors in SY5Y cells**. **A. **Quantification of mRNA in PMA-treated SY5Y cells. Bar legend refers to all panels in figure 2; **B. **magnification of low abundant factors in panel A.; **C. **Modulation of transcription by amitriptyline; **D. **by fluoxetine; **E. **by tranylcypromine; **F. **by reboxetine. Values below 1 indicate antidepressant dependent reduction of growth factors.

In this study, we tested the hypothesis that anti-depressive treatment modulates growth-factor transcription. Antidepressants of different classes were administered to SY5Y cells, which were pre-treated with the phorbol-ester PMA, to induce differentiation into neurons [15,16]. Figure 1B shows that after PMA application for 10 days, most of SY5Y cells developed long processes, while untreated controls did not (Figure 1A). Administration of fluoxetine (Figure 1C) and tranylcypromine (Figure 1D) to PMA-treated SY5Y cells did not induce additional neurite growth, but led to an increase of intracellular granules. The effects of magnetic stimulation and antidepressants on different growth factor transcription were analysed by rtPCR and were summarized in figure 2. Table 1 shows the corresponding p-values.

**Table 1:** Statistical significance analysis

	**2 days**	**7 days**	**14 days**		**2 days**	**7 days**	**14 days**
	NGF				NT-3		
Amitryptiline	< 0.0001	n.s.	n.s.	Amitryptiline	< 0.0001	n.s.	0.0001
Fluoxetine	< 0.0001	n.s.	n.s.	Fluoxetine	< 0.0001	n.s.	0.0017
Tranylcypromine	0.0014	n.s.	0.0032	Tranylcypromine	< 0.0001	n.s.	0.0002
Reboxetine	< 0.0001	n.s.	n.s.	Reboxetine	0.0004	0.0027	0.0001
							
	bFGF				Leptin		
Amitryptiline	0.0012	n.s.	< 0.0001	Amitryptiline	< 0.0001	< 0.0001	0.0003
Fluoxetine	< 0.0001	n.s.	< 0.0001	Fluoxetine	n.s.	< 0.0001	< 0.0001
Tranylcypromine	0.004	< 0.0001	n.s.	Tranylcypromine	n.s.	< 0.0001	< 0.0001
Reboxetine	< 0.0001	n.s.	< 0.0001	Reboxetine	n.s.	0.0021	< 0.0001
							
	CNTF				CREB		
Amitryptiline	n.s.	0.0003	< 0.0001	Amitryptiline	n.s.	< 0.0001	n.s.
Fluoxetine	0.001	< 0.0001	< 0.0001	Fluoxetine	n.s.	n.s.	n.s.
Tranylcypromine	0.002	< 0.0001	< 0.0001	Tranylcypromine	0.0001	< 0.0001	n.s.
Reboxetine	n.s.	< 0.0001	< 0.0001	Reboxetine	< 0.0001	< 0.0001	0.0002
							
	GDNF			--	BDNF		
Amitryptiline	0.001	< 0.0001	0.005	Amitryptiline	n.s.	n.s.	0.002
Fluoxetine	< 0.0001	< 0.0001	0.0003	Fluoxetine	0.0005	0.0012	n.s.
Tranylcypromine	< 0.0001	n.s.	0.0009	Tranylcypromine	n.s.	0.0007	n.s.
Reboxetine	n.s.	n.s.	0.0006	Reboxetine	< 0.0001	n.s.	0.0017

Expressions of growth factors were tested with Student's t-test against the corresponding expression levels at the start of the experiments. Expression levels of controls, which were treated for 10 days with PMA, were normalized before testing. The values in the table represent the p-values, obtained by the t-test. The significance level was set at α = 0.004.

Growth factors became strongly up or down regulated in PMA-treated SY5Y cells during cultivation. Figures 2 A, B show that NGF, NT-3, GDNF and BDNF transcription increased continuously over the 14 day cultivation period upon PMA treatment, while bFGF, CNTF and Creb showed a steady decline, but these changes were compensatory modulated by antidepressant drugs (Figure 2C–F). Moreover, the most abundant growth factors like NGF, GDNF, NT-3 and BDNF, which also showed the strongest increase, were down-regulated by all four antidepressants after 14 days of treatment. By contrast, bFGF and CNTF, which declined upon PMA application, became up-regulated by the drugs, except by amitriptyline. The general common observation was that all four antidepressant drugs counteracted alterations of growth factor transcription, induced by PMA. The factors, which declined upon PMA treatment, became up-regulated by antidepressant drug treatment, while others, which increased were down-regulated. The results of the significance analysis by Student's t-test are displayed in Table 1. It shows that most changes in growth factor expression were significant with regard to the corresponding controls, which have been treated with PMA only.

### Repetitive magnetic stimulation (rTMS)

**Figure 3:**
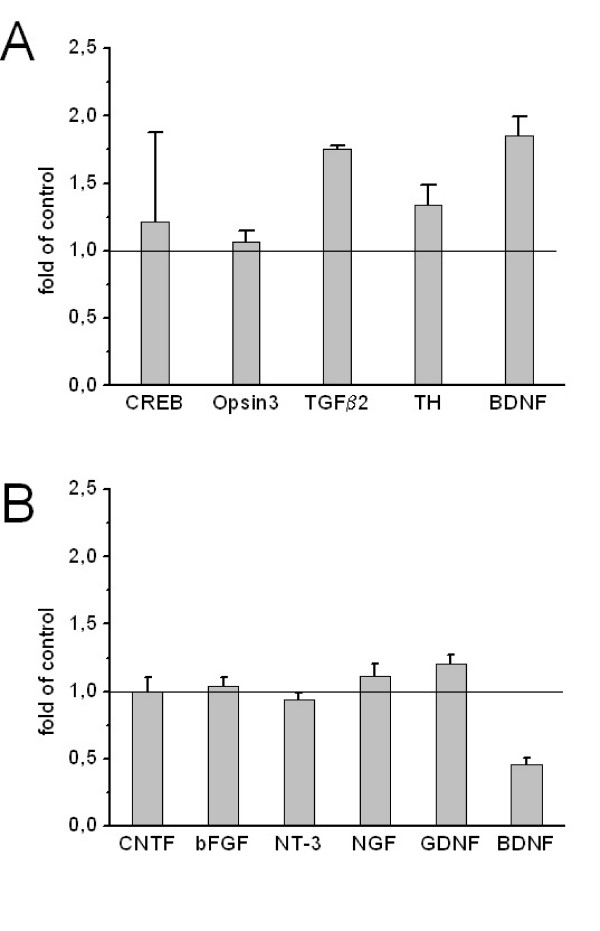
**Effects of rTMS**. Transcription of growth factors after rTMS in **A. **PC-12 cells and in **B. **SY5Y cells. mRNA levels of untreated control cells are set to 1.

A pilot study was conducted to investigate effects of repetitive magnetic stimulation on excitable PC-12 cells. Figure 3A shows that repetitive magnetic stimulation caused an increase of BDNF, TGFβ2 and tyrosine hydroxylase transcription. Undifferentiated SY5Y cells were also subjected to repetitive magnetic stimulation for one week. Figure 3B shows that in contrast to PC-12 cells, where transcription of BDNF was increased, its transcription decreased in SY5Y cells. The results were not significantly different in parametric and non-parametric tests (data not shown), due to small sample numbers.

## Discussion

Because of increasing evidence against the "catecholamine deficiency hypothesis" to explain patho-mechanism of depression, an alternative hypothesis on the mechanism of antidepressants, the "plasticity hypothesis", was proposed [17-19].

In this study we analyzed the transcription of growth factors in cell culture systems. SY5Y cells were pre-treated by the phorbolester PMA to induce differentiation into neuronal cells [15,20]. PMA changed transcription levels of several growth factors massively, but we found that antidepressant drugs counteracted these PMA-induced changes.

Recent post mortem studies in suicide subjects revealed significantly lower levels of BDNF in the prefrontal cortex and hippocampus. Authors of several studies provided evidence that BDNF could be involved in depressive behaviour [21]. Because growth factors induce the formation of neurite-like processes [22,23], we concluded from our microscopic images that the growth factors were also expressed in SY5Y cells. However, we couldn't confirm the expression for every factor (data not shown). The morphological changes provide some evidence that the modulation (up and down regulation) of several growth factors is responsible for the observed effect. A direct correlation with a single growth factor, like BDNF, can not be causal [24]. All four classes of antidepressants, tested in our study, antagonistically reversed strong up or down regulation of growth factors, induced by PMA in cultured SY5Y cells [15,20].

The up-regulation of BDNF and Creb *in vivo *seem to be a central part of the antidepressant properties of antidepressants [17,19,25], however, there is few evidence for the variation of other growth factors so far. Different modes of stress can induce either selective up and down regulation of BDNF splice variants [26] Earlier gene transcription studies in *in vivo *systems had shown that several genes were either up- or down-regulated by antidepressant treatment [27,28]. These and our observations assign a role of modulators to antidepressants, rather than pure stimulators of growth factor transcription.

We found that if transcription of a factor increased during the cultivation period, this increase was attenuated by antidepressants. On the other hand, antidepressants increased the transcription of factors that became down regulated upon PMA-treatment. The antagonistic regulation of growth factors was a common property of all antidepressant classes; however, there were differences in kinetics and specificity. Amitriptyline, for example, did not prevent the PMA-induced decrease of bFGF and CNTF, but it worked antagonistically for all other factors. In general, it favoured more down regulation than other drugs. Reboxetine worked in an exactly opposite way on the transcription of growth factors, by strongly increasing down regulated factors and modestly reducing up regulated factors. Fluoxetine and tranylcypromine represented classes with similar modulator properties, but with different kinetics. Fluoxetine has been shown to increase the expression of bFGF in rats [29]. This was observed in our experiments too.

In a pilot experiment PC-12 cells were treated with rTMS. These cells were chosen, because they are electrical excitable, which made them potentially susceptible for repetitive magnetic stimulation. These cells showed an increase in BDNF, TGFβ2 and tyrosine hydroxylase (figure 3A). In contrast to PC-12 cells, SY5Y cells showed a decrease of BDNF (figure 3B). BDNF mRNA was elevated in all treated samples, compared to controls; however, statistical significance was not reached, due to small sample numbers. It has been shown previously that rTMS and antidepressants acted in a similar way in rat hippocampus [30] and humans [31,32]. We could only partly support this hypothesis.

## Conclusion

In summary, we found that antidepressants normalized PMA-induced growth factor variations and specifically modulated their expression, depending on the type of the drug. We conclude from our experiments that antidepressant drugs rather modulate PMA-induced changes of growth factor transcription, than induce an overall transcription increase. This suggests that a common basic mechanism of antidepressants normalizes pathologic deregulation of growth factors, which without treatment would affect neuronal plasticity.

## Methods

### Cell culture

Cell lines (PC-12 and SY5Y) were obtained from ECACC, Salisbury/UK.

PC-12 cells were grown in RPMI 1640 medium while f cells and SY5Y cells were cultured in Dulbecco's Modification of Eagle's medium, supplemented with 10% foetal calf serum, 100 IU/ml penicillin and 100 mg/ml streptomycin (all reagents from Biochrom, Berlin/Germany) at 37°C in a 5% CO_2 _humidified incubator. 50 % of the medium was exchanged every 3 – 4 days. PC-12 cells were seeded at a density of 40000 per cm^2 ^and SY5Y cells at a density of 10000 per cm^2 ^in 25 cm^2^-flasks coated with 8 μg collagen IV per cm^2 ^(Corning Costar, Bodenheim/Germany; Sigma Aldrich, Deisenhofen/Germany) and allowed to attach for several hours. Cells were collected after trypsinization and washed 3 times with sterile PBS. Primary rat hippocampus cells were isolated and cultured as described earlier [33].

### PMA-differentiation of SY5Y cells and drug application

SY5Y cells were incubated with 16 nM PMA (phorbol 12-myristate 13-acetate) 10 days prior to the experiment. PMA was always renewed when the medium was changed and continued to be present during the course of the experiments. Antidepressant drugs were supplied and renewed every 3 days. Toxic concentrations were determined by the MTT-test (methylthiazolydiphenyltetrazolium bromide) [34]. The drugs were applied at concentration that showed no toxicity in the MTT-test. The concentrations used: amitriptyline 7 μM, fluoxetine 7 μM, tranylcypromine 100 μM and reboxetine 5 μM. All experiments were repeated independently at least 8 times in duplicates.

### Repetitive magnetic stimulation

The flasks were paired and transported to the stimulator. Control cells were placed on a separate table and covered with a blend. Cells to be stimulated were placed on a polystyrene block and a round coil was adjusted directly above. Each treatment consisted of 1000 pulses at an intensity of 0.63 Tesla at 10 Hz, delivered in 20 trains with dwell time of 25 seconds. During the procedure the coil was cooled by ventilation or cooling spray. Afterwards the cells were immediately returned to the incubator. Cells were treated every 12 hours and harvested directly after the 15^th ^treatment.

PC-12 cells were washed twice with PBS prior to trypsin-detachment, centrifuged (500 g, 5 min, 21°C) and washed two more times. The pellet was stored at -20°C. Experiments were repeated two times.

### Real-time PCR

mRNA was extracted with "SV Total RNA Isolation System" (Promega, Mannheim/Germany) PC-12 cells or with the "RNeasy Mini Kit" (Qiagen) for SY5Y according to manufacture's protocol. cDNA was synthesized using iScript cDNA Synthesis Kit (Biorad, Munich/Germany) following manufacturer's instructions. Quantitative PCR was performed with 25 μl reaction mix containing 5 μl cDNA-dilution, 12.5 μl iQ SYBR Green Supermix (Biorad) and 1.5 μl of F- and R-primers for PC-12 cells. 12.5 μl iQ SYBR Green Supermix, 1 μl primer pair, 1 μl cDNA template, 10 μl water. For the sequences of the primer pairs (MWG-Biotech AG, Ebersberg/Germany) see Additional file 1 'Primer pairs'. PCR-amplification was performed on iCycler (Biorad) using a three-step standard protocol with an annealing temperatures of 62°C (human BDNF, CNTF, NGF and bFGF), 60°C (all rat primers) and 55°C (human GDNF, NT-3, Leptin and Creb).

All PCR experiments were done in duplicates. β-actin was used as internal standard and mRNA copies were calculated by the formula: mRNA = 2^-ΔCT^. ΔCT-values were calculated from differences between the respective growth factors and β-actin.

### Data analysis and statistical calculation

For each growth factor, four antidepressants were compared (on day 2, 7, 14) with a control sample. Therefore we used a method for correction for multiple testing (Bonferroni adjustment for each growth factor. Thus, the significance level alpha was set at α = 0.004. All statistical tests were two-sided. The calculations were performed with the statistical package SPSS (SPSS Inc., Chicago, Il).

## Authors' contributions

AWH: Experimental design, writing of the manuscript and supervision of all experiments. WS: Design, writing of the manuscript and supervision of the TMS experiments. AR: Conducted the experiments on antidepression drugs and analyzed the data. UR: Statistical analysis. CR: Conducted the experiments on repetitive magnetic stimulation and analyzed the data. DB: Supervision of PCR and construction of primer pairs. JMM: Supervision of cell culture. JK: Organization, literature, concept and interpretation. JW: Organization, interpretation and supervision. All authors read and approved the final manuscript.

## Supplementary Material

Additional file 1 Primer pairs. Sequences of the primer pairs, used for PCR. Click here for file
