# Supplementary material for: Antidepressant drugs modulate growth factors in cultured cells
Source: BMC Pharmacol. 2008 Mar 4;8:6. doi: 10.1186/1471-2210-8-6 (PMC2275236; doi:10.1186/1471-2210-8-6)
Supplement: Additional file 1 — Primer pairs. Sequences of the primer pairs, used for PCR. [file 1471-2210-8-6-S1.doc]

### Supplemental material

The following primer pairs for rtPCR were used in our experiments:

For PC-12 cells, primers from rat:

TH-F GCT GTC ACG TCC CCA AGG TT

TH-R CAG CCC GAG ACA AGG AGG AG

BDNF-F TCC CTG GCT GAC ACT TTT GAG

BDNF-R ATT GGG TAG TTC GGC ATT GCG

CREB-F AGT GAC TGA GGA GCT TGT ACC A

CREB-R TGT GGC TGG GCT TGA AC

TGFβ2-F GAC GAG GAG TAC TAC GCC A

TGFβ2-R ACT GCT GGG ACT CCA GTC TG

β-actin-F AGG CCA ACC GCG AGA AGA TGA CC

β-actin-R GAA GTC CAG GGC GAC GTAG CAC

For SY5Y cells, primers from human:

BDNF-F AGC TGA GCG TGT GTG ACA G

BDNF-R CGC CAG CCA ATT CTC TTT TTG

Leptin-F TGC GGA TTC TTG TGG CTT TG

Leptin-R GAA TGA AGT CCA AAC CGG TG

GDNF-F GCT GTC TGC CTG GTG CTG CTC

GDNF-R GCC TGC CGA TTC CGC TCT CTT C

B FGF-F GGC TTC TTC CTG CGC ATC CA

B FGF-R GCT CTT AGC AGA CAT TGG AAG A

CNTF-F TGG CTA GCA AGG AAG ATT CG

CNTF-R CCA GGA GTA TCA TTA ACT CCT C

NGF-F AAC AGG ACT CAC AGG AGC A

NGF-R CCT TCC TGC TGA GCA CAC A

NT-3-F CAA CAG AGA CGC TAC AAC TC

NT-3-R CTG AAG TCA GTG CTC GGA C

β-AKTIN-F CTG GAA CGG TGA AGG TGA CA

β-AKTIN-R AAG GGA CTT CCT GTA ACA ATG CA

TGFβ2-F AAA GTG GAC GTA GGC AGC AAT TA

TGFβ2-R GAC CAA CCG GCG GAA GA
